# Senescence, NK cells, and cancer: navigating the crossroads of aging and disease

**DOI:** 10.3389/fimmu.2025.1565278

**Published:** 2025-04-04

**Authors:** Marina Gergues, Rafijul Bari, Sharmila Koppisetti, Anna Gosiewska, Lin Kang, Robert J. Hariri

**Affiliations:** Research and Development, Celularity Inc., Florham Park, NJ, United States

**Keywords:** cellular senescence, NK cells, adoptive immunotherapy, NK cell therapy, cancer

## Abstract

Cellular senescence, a state of stable cell cycle arrest, acts as a double-edged sword in cancer biology. In young organisms, it acts as a barrier against tumorigenesis, but in the aging population, it may facilitate tumor growth and metastasis through the senescence-associated secretory phenotype (SASP). Natural killer (NK) cells play a critical role in the immune system, particularly in the surveillance, targeting, and elimination of malignant and senescent cells. However, age-related immunosenescence is characterized by declining NK cell function resulting in diminished ability to fight infection, eliminate senescent cells and suppress tumor development. This implies that preserving or augmenting NK cell function may be central to defense against age-related degenerative and malignant diseases. This review explores the underlying mechanisms behind these interactions, focusing on how aging influences the battle between the immune system and cancer, the implications of senescent NK cells in disease progression, and the potential of adoptive NK cell therapy as a countermeasure to these age-related immunological challenges.

## 1 Introduction

**Figure 1:**
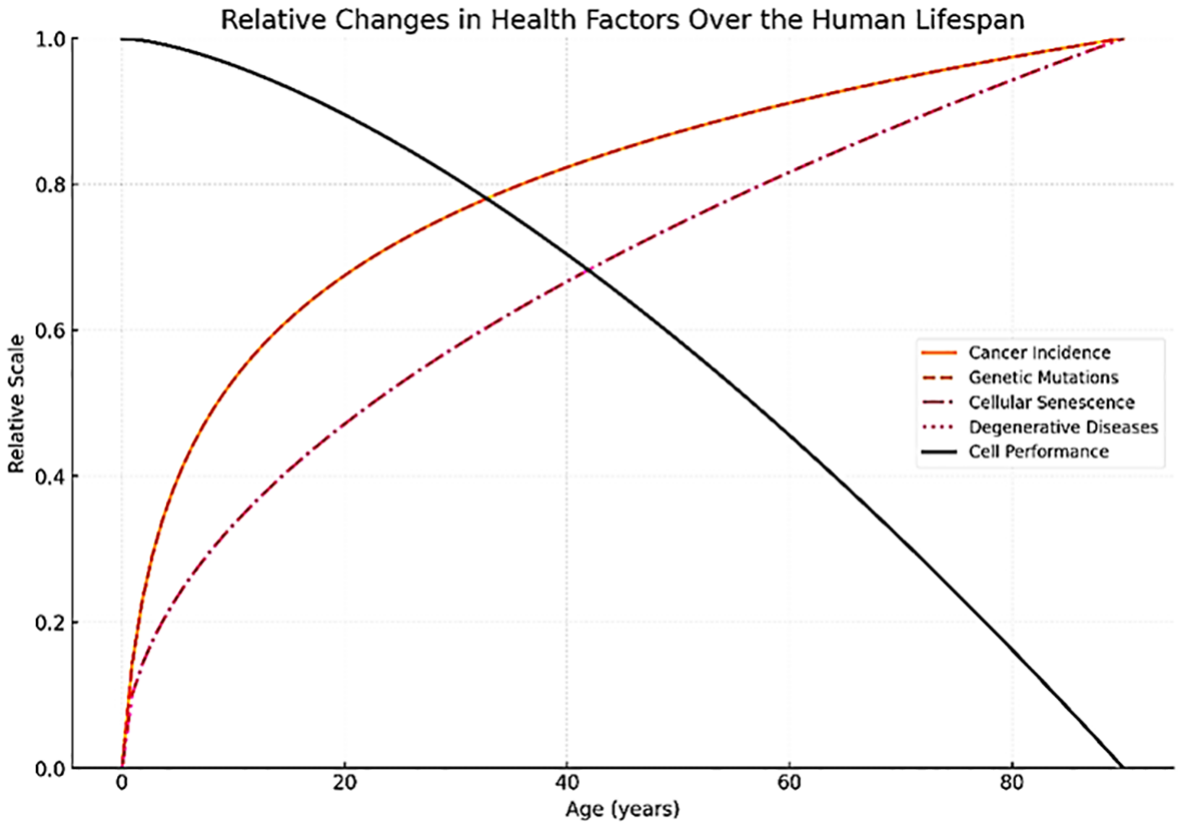
Changes of health factors relative to aging. Aging is associated with increased cellular senescence, age-related diseases, genetic mutations, and cancer incidence, whereas physical fitness progressively declines.

The World Health Organization (WHO) estimates that by 2050, over 2 billion people will be aged 60 or older (1). However, while overall age increases, the number of years in good health remains constant, suggesting that the extended years are often accompanied by illness. As shown in **Figure 1**, aging is associated with cellular senescence, age-related various diseases, genetic mutations, and increased cancer incidence, whereas physical fitness progressively declines. Research indicates that the incidence of chronic diseases such as heart disease, diabetes, neurodegeneration, and cancer increases steeply with older age (2). With this increased prevalence of age-related disease comes associated healthcare costs. For instance, the economic burden of dementia is projected to rise from $263 billion in 2019 to $1.6 trillion by 2050 (3). Similarly, the cancer-attributable costs in the United States are projected to increase from $183 billion in 2015 to $246 billion by 2030 (4). These trends highlight an urgent need for effective healthcare strategies to address the challenges posed by an aging population as part of this demographic shift.

According to the ‘Geroscience Hypothesis’, targeting fundamental aging mechanisms at the molecular and cellular level, rather than treating age-related diseases one by one, could better expand lifespan and reduce the risk of chronic disease (5). These fundamental aging mechanisms, known as the hallmarks of aging, have been well established and include genomic instability, telomere attrition, epigenetic alterations, loss of proteostasis, dysregulated nutrient sensing, mitochondrial dysfunction, cellular senescence, progenitor cell exhaustion, and altered intercellular communication (6). Since these fundamental aging mechanisms are interconnected, the Unitary Theory of Fundamental Aging Processes suggests that interventions targeting any fundamental mechanism might positively influence others (5). For example, addressing cellular senescence could reduce inflammation, improve mitochondrial function, restore proteostasis, and reduce various signs of aging and disease. Given these insights, it’s clear that understanding the interactions between these mechanisms is crucial for developing effective therapeutic interventions.

The relationship between senescence, natural killer (NK) cells, and cancer constitutes a pivotal axis in oncology and gerontology, presenting both challenges and opportunities for therapeutic intervention (7). Cellular senescence, a state of stable cell cycle arrest, emerges as a double-edged sword in cancer biology, acting as a barrier against tumorigenesis in young organisms while potentially promoting tumor growth and metastasis in the aging population through the senescence-associated secretory phenotype (SASP) (8, 9). Further, NK cells, which are critical components of the innate immune system, play a significant role in the surveillance and elimination of malignant and senescent cells, slowing down the progression of cancer and the accumulation of senescent cells that contribute to aging (10). However, the efficacy/integrity of NK cell function diminishes with age, a process known as immunosenescence, which reduces the organism’s ability to fight infections, eliminate senescent cells, and suppress tumor formation. This decline is further complicated by the presence of persistent senescent NK cells, which may contribute to the aged immune phenotype and exacerbate age-related diseases, including cancer (11). Accordingly, preserving the function of NK cells during aging is essential for promoting healthy aging and longevity. Additionally, NK cell-based therapies, particularly adoptive NK cell therapy, demonstrate promise not only in treating cancer and viral infections but also in rejuvenating the aging immune system, eliminating senescent cells (SNCs), and alleviating the effects of SASPs, thereby mitigating the effects of aging and disease progression (12, 13).

This review is structured into four sections: I. Exploring the intricate relationship between aging, senescence, and cancer. II. The impact of senescence on the immune system. III. Highlighting the critical role of NK cells in the removal of senescent cells. IV. An overview of innovative therapeutic strategies, with a particular focus on the potential of adoptive NK cell therapy as an effective solution to age-related issues.

## 2 The shadows of time: exploring senescence in the aging process and cancer

Cellular senescence is a state of stable cell cycle arrest in which cells are resistant to apoptosis and remain metabolically active. Initially discovered in the 1960s by Leonard Hayflick, this cell state has emerged as a pivotal mechanism in aging and cancer (14). Hayflick originally identified senescence in the context of replicative lifespan limits due to telomere shortening. However, it has since been understood to encompass a response to various forms of cellular stress, including genomic damage, oxidative stress, and oncogenic signaling (8, 15). Therefore, this process plays a crucial role as a potent tumor suppressor and is intricately linked to age-related pathologies, playing roles in wound healing and development. The following sections explore the diverse roles of triggers of cellular senescence, markers of cellular senescence, senescence related signaling pathways, and SASP, along with their implications for aging and cancer.

### 2.1 Triggers of cellular senescence

Several factors trigger senescence, which initiates a complex signaling network that ultimately leads to cellular senescence. In this section, we discussed the factors that are known to trigger senescence, including telomere attrition, DNA damage and genomic instability, oncogene activation, and extrinsic triggers.

#### 2.1.1 Telomere attrition

Telomere shortening is considered the most fundamental and well-established trigger of cellular senescence and occurs as cells divide due to the end replication problem (16), which prevents DNA polymerases from fully replicating the ends of linear DNA. Critically short telomeres can mimic DNA breaks, activating DNA damage responses (DDR) like γH2AX phosphorylation (17) and leading to cell cycle arrest mediated by pathways involving ATM, ATR, p53, and p21 (18). This underscores telomeres’ role as a molecular clock for cellular lifespan (19).

Most human somatic cells have limited or no telomerase activity, resulting in telomere erosion with age. Telomere attrition is one of the most direct and predictable pathways to senescence, as evidenced by studies indicating a correlation between telomerase activity and telomere length (TL), which are essential for healthy aging. For instance, research notes a significant decline in telomerase activity in aging lymphocytes, correlating with reduced TL (20).

Further studies show a relationship between leukocyte TL and age-related diseases in various longevity groups, suggesting that parental longevity may influence offspring health and TL maintenance. Super-centenarians experience telomere shortening but maintain higher genome stability than younger individuals, indicating that cellular mechanisms exist to preserve DNA integrity despite aging (21, 22).

Promoting telomerase activity has been identified as a potential strategy for maintaining TL. Increased aerobic activity has been linked to higher telomerase activity and better TL maintenance (23). Similarly, endurance exercise has been shown to support TL maintenance through enhanced telomerase activity (24, 25). In cancer biology, cancer cells often upregulate telomerase or activate alternative lengthening of telomeres (ALT) to maintain TL, which facilitates uncontrolled growth (26–28). In healthy cells, telomerase is typically repressed (29, 30), and its reactivation in cancer cells supports tumor survival by stabilizing TL (31). ALT involves homologous recombination-based telomere elongation, a telomerase-independent pathway for telomere maintenance. ALT activation in somatic cells is rare and poorly understood, highlighting telomere biology’s complexity and its implications for senescence and tumorigenesis (32, 33).

Hormonal regulation impacts telomerase activity, particularly through estrogen, which activates telomerase by affecting the hTERT promoter (34, 35). This is crucial for understanding hormone roles in normal functions and estrogen-related cancer risks. Hormone Replacement Therapy (HRT), often containing estrogen, helps alleviate menopause symptoms and lower osteoporosis and cardiovascular disease risks in postmenopausal women (36–38). Estrogen enhances telomerase activity by binding to estrogen receptors, increasing TERT expression in various cell types (39–42). Clinical studies show that HRT boosts telomerase activity in osteoblasts, promoting bone health (43–45), but it may also elevate cancer risks like breast and endometrial cancer (46). Additionally, antioxidants such as vitamin D and omega-3 fatty acids are linked to increased telomerase activity (47, 48), suggesting a protective effect on telomere length and cellular health.

#### 2.1.2 DNA damage and genomic instability

DNA damage can result from various internal and external factors, such as reactive oxygen species (ROS), ultraviolet radiation (UV), and chemical exposures (49). Accumulation of DNA damage triggers the DNA damage response (DDR), a signaling network that detects damage, halts the cell cycle, and initiates repair. Persistent DDR signaling can lead to cellular senescence (50), a state where cells enter permanent growth arrest, often regulated by proteins like ATM, ATR, and p53 (15).

Cells undergoing senescence can experience cell cycle arrest either in the G1 phase, preventing DNA replication in damaged cells, or the G2 phase, blocking mitosis when DNA is damaged. In the G1 phase, cyclin-dependent kinase (CDK) complexes are essential for transitioning from G1 to S phase by phosphorylating retinoblastoma protein (RB), which regulates growth arrest and proliferation (51). Senescence is often associated with reduced cyclin-CDK activity or increased CDK inhibitors like p21, p15, and p16 (52, 53). Elevated levels of p21 or p16 can induce cell cycle arrest (54) by binding to cyclin E–CDK2 and cyclin A–CDK2 complexes, keeping RB hypo-phosphorylated and sequestering E2F transcription factors (55). Conversely, decreasing CDK inhibitor activity can lead to senescence escape, where cells re-enter the cell cycle (56). In the G2 phase, senescence can occur due to telomere damage, DNA damage, or oncogene activation (17), involving degradation of mitotic regulators by the anaphase-promoting complex/cyclosome (APC/C). Cells unable to progress to mitosis due to inactive cyclin B1–CDK1 complexes enter irreversible arrest. Long-term arrested G2 cells may become tetraploid while remaining blocked before the S phase, associated with RB activation and p16 upregulation (57).

Checkpoints monitor and repair DNA damage during the cell cycle to prevent genomic errors. In the G1 phase, DNA damage activates p53, leading to p21 expression and cell cycle arrest for repair, with apoptosis initiated if damage is irreparable. In the S phase, the ATR-CHK1 pathway stabilizes replication forks to minimize damage. In the G2 phase, the ATM-CHK2 pathway halts the cycle for DNA damage repair before mitosis. Failure to repair DNA can result in mutations and genomic instability, promoting tumorigenesis. Senescence acts as a tumor suppressor by preventing the proliferation of potentially oncogenic cells, primarily through the RB/p16 and p53 pathways (7, 58). Depending on the cellular context, these pathways can work independently or together (54, 59).

The induction of senescence in response to DNA damage acts as a barrier against cancer, highlighting its tumor-suppressive role (60). However, the buildup of senescent cells in tissues contributes to aging and related diseases through the SASP, disrupting tissue homeostasis and promoting chronic inflammation (61).

#### 2.1.3 Oncogene activation

Oncogene-Induced Senescence (OIS) is triggered by the activation of oncogenes such as RAS, BRAF, AKT, E2F1, and cyclin E, or by the inactivation of tumor suppressor genes including PTEN and NF1. This form of senescence acts as a barrier to tumorigenesis by arresting the proliferation of potentially malignant cells (62, 63). Beyond its tumor-suppressive role, OIS has implications in tissue remodeling, organismal aging, and as a target for therapeutic intervention.

Within the intricate web of factors shaping OIS, RB and p53 stand out as pivotal regulators, responsible for halting the cell cycle within senescent cells (64, 65). The upregulation of p53 levels is a hallmark of cells experiencing OIS, which can be disabled when p53 is inactivated. This is demonstrated in experiments with mice genetically modified to carry the oncogenic RasG12D mutation, which developed lung cancer (63). Another key player in this process is p16, a regulator essential for maintaining senescence (54). Lack of p16 allows cells to escape senescence and re-enter the cell cycle. RB, which acts downstream of p16, plays a critical role in enforcing senescence. During senescence, RB specifically targets and represses E2F-targeted genes that are vital for DNA replication (66). Without RB, cells can wrongly commence DNA replication in response to senescence signals, leading to unchecked cell growth and significant genomic instability due to the disruption of a p21-dependent checkpoint (67). This pathway illustrates the delicate balance within cellular machinery, a balance that must be maintained with precision to prevent the onset of cancer by halting the proliferation of potentially harmful cells. As a critical tumor suppressive mechanism, OIS prevents the proliferation of cells that harbor oncogenic mutations. The presence of senescent cells in pre-malignant lesions supports the notion that OIS is an early barrier to tumorigenesis.

#### 2.1.4 Extrinsic triggers

Oxidative stress, resulting from an imbalance between ROS production and antioxidant defenses, can induce senescence by causing DNA, protein, and lipid damage (68). This leads to the activation of DDR and senescence pathways, highlighting the role of antioxidants in modulating cellular response to ROS (69). Stress-Induced Premature Senescence (SIPS) can be triggered by various exogenous stressors, including DNA damage and mitochondrial dysfunction, without the involvement of telomere shortening (70). This indicates a telomere-independent pathway to senescence, underscoring the diversity of triggers that can lead to cellular senescence. Senescent cells secrete various pro-inflammatory cytokines, chemokines, growth factors, and proteases, known collectively as SASP. While SASP can facilitate tissue repair and regeneration, it can also promote chronic inflammation and contribute to the development of age-related pathologies (49, 61). Chronic exposure to mitogenic signals, such as those from aberrant growth factor signaling, can induce a hyperproliferative state that eventually triggers senescence, likely because of DNA damage accumulation and metabolic stress (71). Changes in the epigenetic landscape, including DNA methylation, histone modification, and chromatin remodeling, can trigger senescence. These alterations can affect the expression of genes critical to the cell cycle, DDR, and SASP (72). Extrinsic stressors contribute to cellular senescence, but they are often secondary triggers that accelerate rather than directly initiate the process.

The triggers of cellular senescence play dual roles in health and disease. On one hand, senescence acts as a crucial tumor suppressive mechanism. On the other hand, the accumulation of senescent cells contributes to aging and the development of age-related diseases (73, 74). Research aimed at further dissecting these triggers and their mechanisms will be crucial for harnessing the benefits of cellular senescence while mitigating its adverse impacts on health (75, 76).

#### 2.1.5 Autophagy and senescence

Autophagy and senescence are two forms of cell death that are interconnected in complex ways. Autophagy can both promote and inhibit senescence. On one hand, the accumulation of damaged proteins and organelles within cells can accelerate the senescence process. For instance, autophagy can degrade the nuclear lamina protein LAMINB1 after oncogenic stress by facilitating its transport from the nucleus to the cytoplasm; this process is associated with oncogene-induced senescence and includes the degradation of SIRT1. In the liver, a deficiency in autophagy has been shown to activate Nrf2, a regulator of liver senescence (77). There is also notable evidence that autophagy supplies free amino acids, which are essential for the mTOR complex 1 (mTORC1). This complex is necessary for initiating the synthesis of SASP components. As a result, mTOR activation can occur after the induction of autophagy in senescent cells, as mTOR acts as a downstream effector of autophagy. These functional connections imply that there are regulatory pathways of autophagy present in senescent cells (78). On the other hand, it is widely accepted that basal autophagy plays a protective role in aging and age-related disorders. As autophagy declines with age, it may contribute to the onset of various diseases. Basal autophagy helps maintain homeostasis by preserving stem cell populations and preventing cellular senescence (79). Overall, autophagy is best understood as a regulator rather than a primary driver of senescence.

### 2.2 Hallmarks of cellular senescence and senescence biomarker

Cellular senescence is a dynamic and highly regulated state of cells. Since there are no universal markers for identifying senescence, a set of common characteristics has been established. Herein, we discussed a multifactorial approach to identify and assess cellular senescence, including morphological and structural change-based, molecular, epigenetic, and metabolic markers.

#### 2.2.1 Morphological and structural change-based markers

Senescent cells exhibit distinct morphological characteristics such as enlarged and flat shape, detectable by light microscopy or flow cytometry (80). This increase in cell size is attributed to changes in the cytoskeleton and increased organelle content, including a larger and more complex Golgi apparatus and an expanded lysosomal compartment (81). A significant marker, senescence-associated β-galactosidase (SA-β-gal), reflects increased lysosomal activity, although its specificity has been debated due to activity in non-senescent contexts like neurons and developing embryos (80). Another aging hallmark, lipofuscin, are lipid-containing residues of lysosomal digestion that appear as fine yellow-brown pigment granules. It is commonly referred to as the “age pigment” as it appears in postmitotic cells where it accumulates as a residue of the cell’s metabolic processes (82). Lipofuscin detection by autofluorescence or Sudan Black B staining further delineates senescent cells. Novel approaches, like biotin-conjugated Sudan Black B, enhance the sensitivity of lipofuscin detection, underscoring advancements in identifying senescence-associated structural changes.

#### 2.2.2 Molecular markers

##### 2.2.2.1 DNA damage response markers

Markers including γH2AX, a sign of double-stranded DNA break sites, and DDR protein MRN complex components highlight the DNA damage underlying senescence (83). The recruitment of ATM and ATR protein kinases, and other DDR markers like 53BP1 and MDC1, serve as robust indicators of cellular aging. Furthermore, telomere shortening, measurable via qPCR or FISH, offers insights into replicative cellular aging.

##### 2.2.2.2 Cell cycle markers

An inhibitor of cyclin-dependent kinases, p16INK4a is a critical cell cycle regulator and is often overexpressed in senescent cells (84–86). Its expression is associated with stabilizing the senescent state through the prevention of phosphorylation of Rb. Additionally, the p53 tumor suppressor and its transcriptional target p21 are pivotal in inducing and maintaining senescence, particularly in response to DNA damage (87). Elevated levels of p21 can enforce cell cycle arrest by inhibiting cyclin-dependent kinase activity.

#### 2.2.3 Epigenetic markers

Epigenetic changes, such as the formation of senescence-associated heterochromatin foci (SAHF), indicate senescence through the silencing of proliferation-promoting genes. Detection methods include DAPI staining for SAHFs and immunofluorescence for associated proteins like HP1 and methylated H3K9, offering a window into the epigenetic landscape of senescent cells (88).

#### 2.2.4 Metabolic markers

##### 2.2.4.1 Stress-induced and mitochondrial markers

Oxidative stress, a critical inducer of senescence, leads to ROS production and subsequent DNA damage, pinpointing mitochondrial dysfunction as a pivotal factor (89, 90). Mitochondrial ROS is essential for senescence induction and represents potential biomarkers, which are detectable by flow cytometry. These findings highlight the relationship between oxidative stress, mitochondrial function, and cellular senescence.

##### 2.2.4.2 Increased autophagy and mitophagy

Autophagy is a process where cells degrade damaged or unnecessary cellular components through the lysosomal machinery (91). During cellular senescence, autophagy levels are often elevated as a response to stress and the accumulation of damaged proteins and organelles (79). Markers of autophagy, such as LC3B-II (a lipidated form of LC3 associated with autophagosomes) and a decrease in p62/SQSTM1 (a substrate that is degraded by autophagy), are often used to monitor autophagy levels in cells (92). This process can suppress senescence by removing damaged organelles and proteins, thus preventing the accumulation of deleterious components that would otherwise promote senescence. However, persistent autophagy has been linked to establishing and maintaining the senescent phenotype, suggesting that autophagy can also facilitate senescence under certain conditions. Mitophagy, a specific form of autophagy focused on the selective degradation of mitochondria, serves as a crucial mechanism for eliminating damaged or dysfunctional mitochondria (93). The efficiency of mitophagy decreases with age, and a decrease in mitophagy efficiency is often associated with aging and senescence, highlighting its role as a marker of cellular aging (94).

### 2.3 Key signaling pathways play a crucial role in regulating cellular senescence

Previous discussion has highlighted the importance of the p16INK4a/Rb and p53/p21 pathways as a response to DNA damage-induced senescence. In conjunction with these, a growing body of evidence suggests the mechanistic target of the rapamycin (mTOR) pathway plays a significant role in senescence and aging (95–97). mTOR functions through two major complexes, mTORC1 and mTORC2, each with distinct cellular roles. mTORC1 is known for its involvement in promoting anabolic processes, inhibiting autophagy, and responding to nutrient availability, which can contribute to cellular senescence when dysregulated. mTORC1’s regulation of autophagy is particularly significant in the context of senescence. By inhibiting the initiation of autophagy through the phosphorylation of ULK1, ATG13, and other components of the autophagy machinery, mTORC1 activation can prevent the removal of damaged cellular components, leading to cellular aging. Conversely, the inhibition of mTORC1 has been shown to activate autophagy, potentially delaying the onset of cellular senescence​. mTORC2 also affects cell survival and metabolism and can indirectly influence senescence through its regulation of autophagy and apoptosis​. Its activation by growth factors leads to the activation of Akt, promoting cell survival and proliferation (98, 99). However, the detailed mechanisms by which mTORC2 influences senescence are less understood than those of mTORC1​. Overall, the mTOR pathway’s dual roles in promoting growth and inhibiting autophagy make it a central regulator of cellular senescence.

The NF-κB pathway, essential in stress response, exhibits a complex role in senescence (100–102). It can support both cell survival and aging. Activation occurs through two pathways: canonical, prompted by cytokines, leading to nuclear translocation of NF-κB, and non-canonical, involving p100 processing to p52. NF-κB’s activation in senescence, linked to chronic inflammation, is implicated in tumor development and aging, balancing between inhibiting apoptosis and promoting cellular aging.

The SOCS1 (Suppressor of Cytokine Signaling 1) pathway functions in cellular senescence, primarily through its interaction with the p53 pathway (103). Research has shown that SOCS1 modulates the p53 pathway by affecting the expression of a selective group of p53 target genes such as DDIT3, GADD45B, insulin-like growth factor-binding protein 3 (IGFBP3), PMAIP1 and more, which collectively impact cell cycle regulation, apoptosis, DNA repair and metabolism (104, 105). Specifically, SOCS1 inhibition has been found to influence genes associated with senescence and ferroptosis, a form of programmed cell death different from apoptosis and necrosis (102, 106, 107). Ferroptosis is regulated by oxidative metabolism and involves the accumulation of lipid peroxides to lethal levels. SOCS1 operates by terminating cytokine signaling, essential for controlling cell growth and preventing tumor formation. It achieves this by inhibiting the activity of JAK (Janus kinase), which phosphorylates STAT (Signal Transducer and Activator of Transcription) proteins, thereby preventing their recruitment and activation of gene transcription. Interestingly, aberrant or sustained activation of STAT, specifically STAT5, by oncogenic stimuli can lead to a persistent increase in SOCS1 levels, which in turn can activate p53 through a complex involving ATM and p53 itself. Overall, the SOCS1 pathway’s role in cellular senescence involves a complex interplay with the p53 pathway and other cellular mechanisms, indicating its potential as a target for therapeutic intervention in cancer and age-related diseases.

### 2.4 Senescence-associated secretory phenotype

The SASP encompasses a diverse array of molecules secreted by senescent cells, significantly impacting the surrounding tissue environment and influencing physiological and pathological processes. The secreted products include a variety of cytokines, chemokines (CXCLs), growth factors, proteases, and other molecules like ROS, miRNAs, and extracellular vesicles (EVs). Each of these components reflects and reinforces the senescent state through different mechanisms, influencing neighboring cells and contributing to both physiological and pathological processes.

IL-6 and IL-8 are pro-inflammatory cytokines that serve as key markers and mediators of the inflammatory response associated with SASP (108, 109). These cytokines are not only indicative of the senescent state but also actively promote inflammation, which can lead to the recruitment of immune cells to the site of senescence. While this can aid in the clearance of potentially harmful senescent cells, chronic secretion of these cytokines can contribute to a pro-inflammatory milieu, potentially fostering tumor progression and other age-related pathologies. One study examines the pro-inflammatory profile, particularly the IL-6 and IL-8 ratio, in elderly patients and its association with the SASP (110). This research identified a correlation between increased levels of these cytokines and the proliferation of human breast cancer cells. A separate study explored the paracrine effects of senescent mesenchymal stromal cells, emphasizing the role of IL-6 and IL-8 in promoting inflammation and contributing to the senescence of neighboring cells through the NF-kB signaling pathway (111).

Chemokines are a category of signaling proteins that primarily direct the movement of circulating immune cells to sites of inflammation or injury (112). Chemokines attract immune cells to the senescent cells, facilitating the removal of senescent cells. However, similar to IL-6 and IL-8, prolonged chemokine production can contribute to chronic inflammation and disrupt tissue homeostasis, promoting various pathological states (113). Monocyte Chemoattractant Protein-1 (CCL2) is a chemokine that attracts monocytes, memory T cells, and dendritic cells to the site of tissue damage or senescence (114, 115). It is involved in recruiting immune cells to clear senescent cells and has been implicated in promoting both beneficial and deleterious effects, depending on the context. A recent study found that CCL2 secreted by senescent fibroblasts recruited monocytes that released prostaglandin E2, suppressing resident memory T cell activation and proliferation (115). Interestingly, treating older adults with a p38 mitogen-activated protein kinase inhibitor before the challenge diminished CCL2 expression and monocyte recruitment, reducing prostaglandin E2 secretion and thereby enhancing the skin’s response to the viral challenge that caused senescence. CXCL1 is involved in neutrophil recruitment and can be upregulated in senescent cells. It plays a role in wound healing and in the development of chronic inflammation associated with aging and cancer (116, 117). CXCL10 (IP-10) is secreted by several cell types in response to IFN-γ and is involved in the recruitment of T cells, NK cells, and macrophages (118, 119). Its expression in senescent cells contributes to the establishment of an inflammatory microenvironment. A recent study found that senescent hepatocytes release higher levels of CXCL10, which significantly increased NK cell migration, activation and cytotoxicity via the CXCR3 receptor. CCL20 attracts lymphocytes, including T cells and B cells, and has been shown to be part of the SASP (120–122). Its involvement in immune cell recruitment stresses its potential role in both tumor suppression and promotion.

In addition to chemokines, MMPs are enzymes that break down extracellular matrix proteins, playing a crucial role in tissue remodeling and repairing. In senescence, MMPs such as MMP-1, MMP-3, and MMP-10 are secreted as part of the SASP, reflecting the cell’s role in altering its microenvironment (123, 124). By degrading the extracellular matrix, MMPs facilitate tissue remodeling but can also contribute to the invasive potential of neighboring pre-cancerous or cancerous cells by altering the tissue architecture and promoting tumor metastasis.

Growth factors within the SASP, such as Transforming Growth Factor-β (TGF-β), Insulin-like Growth Factor Binding Proteins (IGFBPs), and Vascular Endothelial Growth Factor (VEGF), play pivotal roles in modulating the surrounding tissue environment. Initially, SASP acts as a tumor suppressor by recruiting immune cells to eliminate pre-cancerous cells. However, in advanced cancers, SASP can promote tumor progression by supporting the proliferation of cancer cells (125)​​. Furthermore, SASP factors contribute to chronic inflammation, a hallmark of aging known as “inflammaging.” This chronic, low-grade inflammation is associated with the progression of many age-related diseases, such as cardiovascular diseases, Alzheimer’s disease, and diabetes.

In cardiovascular disease, the secretion of SASP factors can influence the structure and function of blood vessels, contribute to the development of atherosclerotic plaques, and promote inflammation within the cardiovascular system (126). For example, the secretion of pro-inflammatory SASP factors can induce a chronic inflammatory state, contributing to the development of atherosclerosis. These changes impair the ability of blood vessels to accommodate blood flow, increasing the heart’s workload.

Although the connection between SASP and neurodegenerative diseases is an emerging area of research, the inflammatory nature of it may contribute to the pathogenesis of diseases like Alzheimer’s and Parkinson’s. Senescent microglia have been identified as a major contributor to brain aging, particularly in white matter, due to their limited repopulating capacity and susceptibility to aging effects. Further research in this area may lead to novel strategies for delaying or preventing the onset of neurodegenerative diseases.

Senescent cells attract and activate various immune cells, including macrophages, dendritic cells, T lymphocytes, and neutrophils, through the secretion of SASP chemokines (e.g., MCP-1, MIP1-α, RANTES, RARRES2) and cytokines such as IL-6 and TNFα, as well as extracellular mitochondrial DNA and microRNAs (127, 128). These factors facilitate the immune-mediated clearance of senescent cells under normal conditions (129, 130). However, when the burden of senescent cells surpasses a certain threshold, these cells can interfere with the immune system’s ability to clear them. IL-6, for instance, has been shown to impair macrophage migration, a critical process for effective senescent cell clearance (131). Moreover, MMPs secreted by senescent cells can cleave FAS ligands and other immune cell surface proteins, further hindering immune function (132). Additionally, senescent cells can induce fibrosis, creating physical barriers that impede immune cell infiltration and promote the retention of immune cells within inflammatory sites (133). They also express “don’t eat me” signals, such as CD47, which inhibit phagocytosis by macrophages (134).

Research has demonstrated that the number of senescent cells required to induce frailty, limit health span, and cause premature death is higher in young mice compared to old mice and in young lean mice compared to young obese mice. This difference is attributed to the higher pre-existing burden of senescent cells in old and obese mice (135, 136). The abundance of pre-senescent and senescent adipose progenitor cells remains low until early old age in mice, after which it shows a significant increase (137). This upward inflection in older mice supports the idea of a threshold burden that, when crossed, leads to the detrimental effects associated with senescent cell accumulation. Additionally, the lag between chemotherapy-induced senescence and the onset of age-related morbidities is longer in childhood cancer survivors compared to adults who received higher doses of chemotherapy for bone marrow transplantation. This finding suggests that a higher senescent cell burden in adults leads to quicker manifestation of age-related diseases (138). These findings suggest that maintaining the burden of senescent cells below a certain threshold could allow the immune system to effectively clear these cells, potentially extending health span and mitigating age-related diseases.

## 3 Unraveling the mysteries of immunosenescence: the impact of aging on our immune system

Immunosenescence refers to the gradual deterioration of the immune system associated with aging, leading to increased vulnerability to infections, diminished response to vaccination, and a higher risk of developing cancer (139). This process affects both the innate and adaptive components of the immune system, resulting in a compromised ability to respond to pathogens and to internal threats such as tumorigenesis. The significance of immuno-senescence extends beyond individual health, impacting public health policy and healthcare strategies for the aging population.

### 3.1 Myeloid cells

Myeloid cells are a diverse group of cells originating from myeloid progenitor cells in the bone marrow (140). They play crucial roles in both innate and adaptive immunity, as well as in maintaining homeostasis within the body. These include monocytes, dendritic cells (DC), granulocytes, and megakaryocytes. These cells are essential for the body’s defense against pathogens, removing cellular debris, and regulating inflammation. Moreover, they are involved in the pathogenesis of numerous diseases, including infections, autoimmune disorders, and cancer.

Aged myeloid cells exhibit an increased propensity for producing pro-inflammatory cytokines, such as IL-6 and TNF-α, in response to stimuli, a phenomenon that has been linked to enhanced NF-κB signaling pathway activity driven by DNA damage and genomic instability (141, 142). This dysregulation of inflammatory responses in aged myeloid cells is implicated in the pathogenesis of several age-related conditions, including atherosclerosis, type 2 diabetes, and neurodegenerative diseases, where chronic inflammation plays a key pathophysiological role (143).

The efficiency of myeloid cells in pathogen recognition and clearance declines with age, contributing to increased susceptibility to infections in the elderly. Studies have shown that macrophages from aged individuals exhibit altered signaling pathways, including reduced responsiveness to IFN-γ, a key activator of macrophage antimicrobial activities (144, 145). In addition, aged DCs display altered cytokine production profiles, reduced expression of costimulatory molecules, and impaired migration to lymph nodes, further compromising the immune response to pathogens (146). Finally, Neutrophils, the first line of defense against infections, exhibit diminished chemotaxis, phagocytosis, and intracellular killing capacity with age. These changes contribute to the increased incidence and severity of infections observed in older adults (147, 148).

Aged macrophages exhibit impaired cholesterol efflux and increased secretion of pro-inflammatory cytokines, promoting plaque formation and instability (149). Additionally, these cells ROS and MMPs, which further exacerbate vascular inflammation and damage (150).

Neurodegenerative disorders, such as Alzheimer’s disease (AD) and Parkinson’s disease (PD), have also been linked to the pro-inflammatory activity of aged myeloid cells. In AD, microglia, the resident myeloid cells of the brain, become hyperactive and release inflammatory cytokines, contributing to neuroinflammation and neuronal damage. This inflammatory response is thought to exacerbate amyloid-beta accumulation and tau pathology, key features of AD (151). Similarly, in PD, activated microglia contribute to the degeneration of dopaminergic neurons by producing pro-inflammatory mediators (152).

Chronic inflammation driven by aged myeloid cells plays a pivotal role in cancer development and progression. Tumor-associated macrophages (TAMs) have been shown to support tumorigenesis by secreting growth factors, cytokines, and proteases that facilitate tumor cell invasion, angiogenesis, and suppression of anti-tumor immunity (153). Furthermore, aged myeloid cells may contribute to the development of cancer-associated cachexia, a syndrome characterized by weight loss and muscle wasting, through the systemic release of inflammatory cytokines (154). These findings underscore the urgent need for further research in this area.

### 3.2 NK cells

NK cells play a pivotal role in early defense mechanisms against infections and in eliminating malignant cells. Aging, a process we all inevitably face, is associated with an increase in the proportion of CD56dim NK cells, which are more mature and possess greater cytotoxic capabilities, and a concurrent decrease in CD56bright NK cells, which are typically less mature and have higher cytokine-producing capacity (155, 156). Furthermore, aged NK cells exhibit a reduction in the expression of natural cytotoxicity receptors (NCRs) such as NKp30, NKp46, and NKp44, which are crucial for recognizing and killing tumor and virus-infected cells (157). These phenotypic changes can impair the ability of NK cells to respond effectively to infections and malignancies.

Functionally, NK cells from elderly individuals show a decrease in their cytotoxic activity. This decline is attributed to various factors, including reduced granule exocytosis and impaired signaling pathways essential for NK cell activation (158). The production of cytokines and chemokines by NK cells, such as IFN-γ, which plays a critical role in enhancing the immune response against tumors and viral infections, is also diminished with age (159). These functional impairments compromise the ability of NK cells to protect against diseases effectively.

Certain cellular stressors and metabolic processes contribute to the immunosenescence of NK cells. Similar to other cells, telomere shortening is evident in NK cells, contributing to decreased proliferative potential and cytotoxic function. In addition, under continuous stress conditions, NK cells secrete the pro-inflammatory SASP that modulate the immune response and alter the tissue microenvironment. Aging in NK cells is also associated with metabolic reprogramming, which affects their energy production and biosynthesis pathways, crucial for maintaining cellular functionality. Changes in metabolic pathways, including glycolysis and oxidative phosphorylation, can influence NK cell activation, proliferation, and cytotoxicity. For instance, aged NK cells exhibit altered mitochondrial function, which can lead to decreased ATP production and increased production of ROS, further contributing to cellular senescence and dysfunction (160, 161).

The decreased cytotoxicity of NK cells in older individuals has been extensively documented and is attributed to various cellular mechanisms. Notably, the process of degranulation, essential for the delivery of cytotoxic granules (containing perforin and granzymes) to target cells, is impaired in aged NK cells. Perforin forms pores in the target cell membrane, allowing granzymes to enter and initiate apoptosis. Studies have shown that aged NK cells have reduced perforin and granzyme B expression, which are crucial for their killing function (159, 162). The molecular basis for the observed decrease in degranulation and cytolytic granule content in aged NK cells involves alterations in signaling pathways and the expression of activation receptors. For instance, the expression of the signaling lymphocyte activation molecule (SLAM) family receptors, which play roles in NK cell activation and cytotoxicity, is altered in aging (163). Furthermore, changes in the lipid composition of the NK cell membrane with age can affect the organization of signaling molecules and the formation of the immunological synapse, thereby impairing degranulation (164).

Studies have highlighted a decrease in the ability of NK cells from older individuals to produce IFN-γ and other cytokines in response to stimulation. Campos et al., indicated that aging impacts the immune system’s functionality, with specific reference to NK cell activity (165). Further, Solana et al.; and Hazeldine et al.; have expanded on this, reporting that the age-associated decline in NK cell function includes not only reduced cytotoxicity but also impaired cytokine production, which is critical for the effective orchestration of the immune response (11, 158). Moreover, the decreased expression of activating receptors and changes in the cellular metabolic profile may affect the ability of NK cells to respond to stimulation and produce cytokines efficiently (166, 167).

Sarcopenia, characterized by the loss of muscle mass and strength, is a reliable model to assess the effects of aging and the associated decline in NK function. Adipose tissue gain is common with aging and poses significant health risks, particularly visceral fat, which is linked to insulin resistance, type 2 diabetes, cardiovascular disease, dementia, cancer, and increased mortality. Obesity can present normal, increased, or decreased muscle mass, but muscle infiltration with fat can mask true muscle health. Obesity may prevent muscle gain in response to exercise, possibly due to skeletal muscle mTOR hyperactivation. The interrelation between sarcopenia, obesity, and immunity in aging involves cytokines produced by adipose tissue (adipokines) and skeletal muscle (myokines) and may contribute to higher mortality and disease susceptibility in the elderly. Studies indicate that in elderly women, higher BMI correlates inversely with NK cell percentage and directly with NK cell apoptosis rate, relationships not observed in young women (168). Therefore, researchers have proposed that muscle wasting and increased adipose tissue in aging lead to decreased IL-15 and increased inflammatory adipokines, negatively affecting NK cells (168).

### 3.3 T cells

T cell immunosenescence is particularly impactful due to T cells’ central role in adaptive immunity. Immunosenescence affects innate and adaptive immune systems, but T cell alterations are among the most studied due to their critical functions in pathogen defense, cancer surveillance, and vaccine responses (169, 170). The thymus, where T cells mature, begins to atrophy early in life (171). The primary consequence of thymic involution is a decreased output of naïve T cells, leading to a narrowed T cell receptor (TCR) repertoire. Moreover, the existing T cell population undergoes homeostatic proliferation to maintain peripheral T cell numbers, accelerating the accumulation of memory T cells and contributing to the T cell senescence phenotype (172, 173). These senescent T cells exhibit reduced proliferative capacity, altered cytokine production, and diminished antigenic diversity. The accumulation of senescent T cells compromises the body’s defense mechanisms, leading to increased susceptibility to novel and re-emerging infections, leaving elderly individuals with higher mortality rates from infectious diseases (111, 174). This process also diminishes vaccine efficacy in the elderly, partly due to the reduced diversity of the T cell repertoire (175). Finally, senescent T cells have impaired surveillance against tumor cells, thereby contributing to a higher incidence of cancer and autoimmune diseases (176).

Aging is associated with a shift from naïve to memory T cells, particularly an accumulation of terminally differentiated effector memory T cells (TEMRA) that re-express CD45RA (177). These cells have been linked to chronic viral infections and are considered at the end of differentiation with limited proliferative capacity but high cytotoxic potential (178). There is also an increase in regulatory T cells (Tregs). This increase has been associated with a higher propensity for immunological tolerance, potentially impairing immune surveillance against tumor cells and contributing to autoimmunity (179, 180). Emerging therapies aim to modulate T cell populations to rejuvenate the aging immune system. Approaches such as low-dose interleukin-2 therapy to selectively expand Tregs in autoimmunity or checkpoint inhibitors to reduce Treg-mediated suppression in cancer are under investigation (181, 182). With aging, T cells exhibit diminished TCR signaling efficiency, which can be attributed to changes in the expression levels and phosphorylation status of key signaling molecules. For instance, the decreased expression of CD3ζ, a component of the TCR complex, and alterations in the Lck and ZAP-70 kinases have been noted in aged T cells (183). These changes impair the T cells’ ability to transduce activation signals following antigen recognition effectively. The cytokine production profiles of T cells also change with age. There is a noted shift towards a more inflammatory cytokine milieu, including increased levels of IL-6 and TNF-α, which are associated with “inflammaging” (184). Changes in cytokine receptor expression further complicate the immune response in the elderly. For example, the altered expression of IL-2 and IL-7 receptors contributes to reduced T cell proliferation and survival, impacting the maintenance of T cell homeostasis (185).

The reduced diversity and function of T cells in the elderly contribute to a higher incidence and severity of infectious diseases (173). Influenza poses a significant threat to the elderly, who are at a higher risk of severe disease and mortality. Studies have shown that reduced T cell responses to influenza vaccination in the elderly correlate with decreased vaccine efficacy, highlighting the impact of T cell immunosenescence (186). The COVID-19 pandemic has highlighted the vulnerability of the elderly to respiratory viruses. Older individuals are more likely to develop severe diseases and complications from SARS-CoV-2 infection. Research has identified impaired T cell responses in aged COVID-19 patients, including a reduced frequency of specific T cell subsets and functional anomalies (187, 188).

### 3.4 B cells

B lymphocytes (B cells) are a part of the adaptive immune system and crucial for humoral immunity (189, 190). B cell immunosenescence is characterized by alterations in B cell subsets, reduced repertoire diversity, and changes in antibody production and quality. These changes impair the immune system’s ability to respond to pathogens and vaccination, influencing the health and well-being of older adults.

Aging is associated with a significant shift in B cell subsets, marked by a decrease in naïve B cells and an increase in memory B cells and age-associated B cells (ABCs) (189). This accumulation of memory B cells, which, while beneficial for rapid responses to previously encountered antigens, limits the immune system’s capacity to respond to new infections and vaccines (191). The expansion of ABCs, characterized by distinctive phenotypic markers such as CD11c and T-bet expression, represents a significant deviation from the normal B cell repertoire, potentially contributing to dysregulated immune responses in the elderly. The age-related increase in ABCs and the resultant shift in the B cell repertoire have been linked to enhanced autoantibody production (192) and correlates with the increased prevalence of autoimmune conditions in older adults (193). The pro-inflammatory cytokines produced by ABCs and other immune cells exacerbate this condition, which is associated with various age-related pathologies, including cardiovascular diseases, type 2 diabetes, and neurodegeneration (194). The diversity of the B cell receptor (BCR) repertoire decreases with age, limiting the range of antigens that can be recognized (195–197). The diminished thymic output contributes to a narrower pool of B cells capable of responding to new antigens (198).

The impaired production of high-affinity antibodies and the decreased B cell repertoire diversity contribute to older adults’ increased susceptibility to infections, including respiratory viruses and bacteria causing pneumonia.

## 4 Cellular rejuvenation: the impact of NK cells on senescent cell clearance

NK cells, essential players in innate immunity, offer the first line of defense against a wide range of pathogens and play a critical role in the detection and elimination of cancerous cells (199, 200). Unlike adaptive immune cells, NK cells do not require prior sensitization to recognize and attack their targets. This immediate response is mediated through a sophisticated network of activating and inhibitory receptors, which enables NK cells to differentiate between infected or malignant cells and healthy cells (10).

NK cells originate from hematopoietic stem cells in the bone marrow and undergo a series of differentiation and maturation stages guided by a complex interplay of cytokines and transcription factors (201). They are not a homogeneous population but consist of various subsets distinguished by their surface receptor expression, functional capabilities, and tissue localization. The classification of these subsets and their distinct roles in immune responses, from cytokine production to direct cytotoxicity, are discussed (202). A delicate balance between signals governed the ability of NK cells to identify and kill target cells from activating and inhibitory receptors. Upon activation, NK cells employ several mechanisms to kill target cells, including releasing cytotoxic granules containing perforin and granzymes and engaging death receptor pathways (203).

Chemokines CCL2 and CXCL8 serve as chemotactic signals that guide NK cells to senescent cells. CCL2 is known to bind to its receptor CCR2 on NK cells, facilitating their migration toward high CCL2 concentrations typically found in the senescent cell milieu (204). Similarly, CXCL8 interacts with CXCR1 and CXCR2 receptors on NK cells, further directing their migration to the sites of senescence (205). The orchestrated action of these chemokines ensures that NK cells are efficiently recruited to areas where their surveillance and cytotoxic functions are required. In addition to chemokines, NK cell migration is influenced by tissue-homing receptors, which are regulated by environmental signals and enable NK cells to reach specific tissues where senescent cells accumulate. The expression of selectins, integrins, and other adhesion molecules on NK cells facilitates their trafficking and extravasation into tissues undergoing senescence or inflammation (206). For instance, the interaction between the integrin LFA-1 on NK cells and its ligand ICAM-1, which can be upregulated on senescent cells or within the senescent tissue microenvironment, is crucial for NK cell tissue infiltration (207). Finally, SASP not only serves to attract NK cells but also modulates their effector functions. Recent studies suggest that specific SASP components can enhance the cytotoxic activity of NK cells. For example, IL-15 is a potent stimulator of NK cell proliferation, survival, and activation (208). This suggests that the SASP can prime NK cells for enhanced recognition and elimination of senescent cells, in addition to recruiting them to sites of senescence.

**Figure 2:**
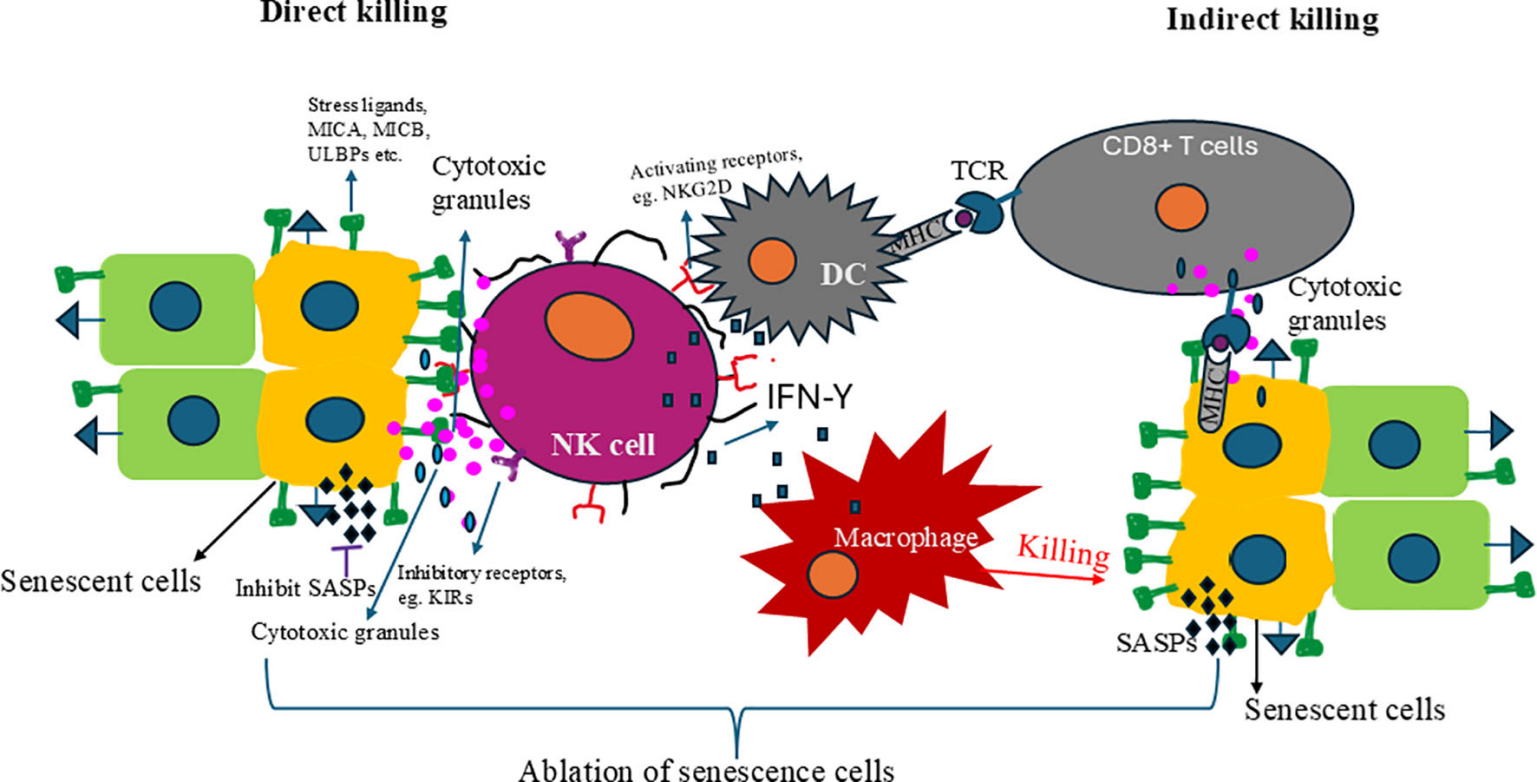
The mechanism of NK cells to remove senescent cells. There are two mechanisms NK cells can use to target and clear senescent cells: direct killing and indirec killing. In direct killing, the NK cell activation receptor recognizes stress ligands whose expressions are upregulated in senescent cells and eliminates them. Those activated cells also release cytokines like IFN-γ, which activate dendritic cells and macrophages that clear the senescence cells. Dendritic cells present senescence-specific antigens CD8+ T cells and activate them. The activated CD8+ T cells recognized the senescence cells and cleared them. Removing senescence cells alleviates senescence-associatec secretory phenotypes (SASPs) and rejuvenates and restores immunity.

The NKG2D is an activating receptor of NK cells that recognizes a variety of ligands, including MHC class I chain-related proteins A and B (MICA and MICB) and the UL16-binding proteins (ULBPs). These ligands are typically upregulated on cells undergoing stress or transformation, such as senescent cells, making them targets for NK cell-mediated cytotoxicity (209, 210). Upon ligand recognition and activation, NK cells mainly use two mechanisms to eliminate senescence cells (SNCs): direct and indirect killing (**Figure 2**). In direct killing, NK cells activating receptor NKG2D can recognize stress ligands like MICA, MICB, and ULBPs known to upregulate in senescent cells, activating NK cells. The activated NK cells release cytolytic granules and cytokines. Released cytolytic granules like perforin and granzyme lyse the SNCs, and cytokines like IFN-Y activate dendritic cells and macrophages (**Figure 2**). The activated macrophages kill the senescence cells, and activated dendritic cells present epitopes to activate T cells, eliminating the senescence cells. Studies have further elucidated the mechanisms underlying the upregulation of these stress ligands in response to cellular stress and damage, highlighting the role of DDR pathways and oncogenic signaling in promoting their expression on the cell surface (211, 212). The balance between activating and inhibitory signals is critical for NK cell response. MHC class I molecules generally provide inhibitory signals to NK cells through their interaction with killer-cell immunoglobulin-like receptors (KIRs), preventing the destruction of healthy cells. This delicate balance, which is a testament to the immune system’s complexity, is crucial for the effective function of NK cells. However, senescent cells often exhibit altered expression of MHC class I molecules, reducing the inhibitory signals and making them more susceptible to NK cell recognition and elimination (213). This alteration in MHC class I expression is a key factor in the “missing-self” hypothesis, which suggests that cells lacking sufficient MHC class I molecules become targets for NK cell-mediated lysis (214). Further, components of the SASP can directly or indirectly influence NK cell response, either by upregulating ligands for activating receptors such as MICA and MICB on target cells, enhancing NK cell activity and proliferation by IL-15, or by modulating the expression of MHC class I molecules and other “self” markers (215, 216).

The interaction between Fas ligand (FasL) on NK cells and the Fas receptor on senescent cells is another pathway leading to apoptosis (217). Furthermore, cytokines produced by NK cells, such as IFN-γ, play a crucial role in inhibiting proliferation and inducing apoptosis in target cells (218). This comprehensive approach of NK cells in eliminating senescent cells reassures us about the effectiveness of the immune system. Despite these mechanisms, some senescent cells can evade NK cell-mediated clearance through various strategies, including the downregulation of activating ligands or the secretion of immunosuppressive components of the SASP (219).

Like cancer cells, senescent cells can alter the expression of surface ligands necessary for NK cell recognition. They can decrease the expression of NKG2D ligands through various mechanisms, thereby reducing their visibility to NK cells. This evasion strategy involves transcriptional downregulation of ligand genes and the shedding of ligands from the cell surface. The latter process is mediated by metalloproteinases, which cleave membrane-bound ligands, releasing them in a soluble form that can bind to NKG2D receptors decoy, leading to receptor downmodulation (220). Additionally, senescent cells express decoy receptors that bind to and sequester activating ligands, preventing engagement with NK cell receptors. This not only prevents the ligands from engaging with their cognate-activating receptors on NK cells but may also lead to the depletion of these ligands in the microenvironment (211). Soluble NKG2D ligands, such as MICA and MICB, are cleaved from the cell surface and bind to NKG2D, leading to receptor downmodulation and impaired NK cell function (221). Furthermore, senescent cells can upregulate the expression of specific “self” molecules, like HLA-E, which bind to the inhibitory receptor NKG2A on NK cells, providing inhibitory signals that counteract activating signals and protect senescent cells from NK cell-mediated killing (222).

The SASP of senescent cells includes a variety of cytokines, chemokines, and growth factors, some of which can have immunosuppressive effects. For example, senescent cells can produce factors like TGF-β and interleukin-10 (IL-10), suppressing NK cell activity and reducing their cytotoxic response towards senescent cells. TGF-β can inhibit NK cell activity by downregulating the expression of NK cell activating receptors, such as NKG2D, and impairing the production of key cytokines like IFN-γ (223). This results in diminished NK cell-mediated cytotoxicity against target cells, including senescent cells. Additionally, TGF-β has been shown to promote the conversion of effector T cells into regulatory T cells (Tregs), further suppressing immune responses (224). IL-10 is well recognized for its ability to inhibit the function of immune cells, including NK cells. It can suppress the cytotoxic activity of NK cells by downregulating the expression of activating receptors and inhibiting the secretion of pro-inflammatory cytokines such as IFN-γ (225, 226). The presence of IL-10 in the tissue microenvironment can thus create an immunosuppressive milieu that protects senescent cells from NK cell-mediated clearance. SASP factors can also recruit and activate immune regulatory cells, such as Tregs and myeloid-derived suppressor cells (MDSCs), which further create an immunosuppressive microenvironment. This can inhibit the activity of NK cells and other effector immune cells, aiding in the evasion of immune surveillance (227). MDSCs are a heterogeneous population of cells that arise from myeloid progenitors and have potent immunosuppressive activities. They can suppress the activities of NK cells and cytotoxic T lymphocytes (CTLs) by producing arginase, nitric oxide, ROS, and cytokines like IL-10 and TGF-β. SASP factors, such as IL-6, IL-1β, and VEGF, have been implicated in the recruitment and activation of MDSCs within the tumor microenvironment or sites of tissue senescence, further contributing to immune evasion (228, 229).

Senescent cells contribute to the remodeling of the ECM by secreting various MMPs, fibronectin, and collagen, which can lead to increased tissue stiffness and the development of fibrotic lesions (230). This fibrotic tissue can act as a physical barrier, impeding the infiltration and mobility of immune cells within the tissue. For instance, increased collagen deposition has been shown to limit T cell penetration into tumor microenvironments, a phenomenon that is likely applicable to areas with high concentrations of senescent cells (231). The secretion of ECM components by senescent cells also leads to alterations in the biochemical properties of the tissue microenvironment. These changes can affect the expression of adhesion molecules and chemokines, further modulating the recruitment and retention of immune cells. For example, the altered ECM can influence the gradients of chemokines required for the guided migration of immune cells, such as NK and T cells, thereby affecting their ability to locate and eliminate senescent cells (232).

These mechanisms contribute to the ability of senescent cells to evade clearance by NK cells, allowing their accumulation in tissues, which is associated with chronic inflammation, tissue dysfunction, and various age-related pathologies. Understanding the interactions between senescent cells and the immune system, particularly NK cells, is crucial for developing therapies aimed at targeting senescent cells to improve age-related diseases and promote healthy aging.

## 5 Therapeutic strategies: innovative approaches to overcoming cellular senescence and boosting NK cell activity

The preventive approach to aging involves understanding the biological mechanisms of aging and intervening early to slow down or reverse these processes. By treating aging as a “treatable” condition, the aim is to extend the healthspan, the period of life spent in good health and free from chronic diseases, rather than just prolonging life without quality.

Senolytic drugs work in senescent cells by selectively inducing apoptosis. These drugs target specific pathways like the BCL-2 family, p53, and the PI3K/AKT pathway, crucial for the survival of senescent cells (233). The elimination of these cells has shown potential in reducing the burden of age-related diseases, including Alzheimer’s disease, cardiovascular diseases, and cancers, thereby significantly improving the quality of life for older adults (234).

One of the first senolytic combinations discovered, Dasatinib (a tyrosine kinase inhibitor) and Quercetin (a flavonoid with anti-inflammatory properties), have shown promising results in preclinical models. A landmark study reported the effectiveness of Dasatinib plus Quercetin in decreasing senescent cell abundance in humans, specifically in a clinical trial involving individuals with diabetic kidney disease. This open-label Phase 1 pilot study demonstrated the potential of senolytic drugs to decrease senescent cell abundance, marking a significant step forward in senolytic therapy research (235). Targeting the inflammatory milieu through the administration of anti-inflammatory drugs, such as metformin or rapamycin, has also shown promise in reducing the deleterious effects of senescent cells on tissue homeostasis and function (236, 237). Clinical trials investigating SASP inhibitors, like the IL-1β inhibitor canakinumab, have shown reduced incidence of lung cancer, highlighting the potential of this approach (238). These strategies, aiming to either suppress the SASP or enhance the immune system’s capacity to eliminate senescent cells, offer a two-pronged approach to combating the negative consequences of cellular senescence.

While senolytic drugs offer a novel approach to treating age-related diseases, they also present challenges, particularly regarding their long-term effects and potential off-target impacts. Most senolytic drugs were initially developed for other purposes and repurposed for targeting senescence, necessitating thorough evaluations to ensure their safety and efficacy for long-term use (239). Additionally, ongoing clinical trials aim to assess the effects of senolytic drugs on various age-related conditions, offering hope and a path toward clinical translation (240).

Combining senolytic drugs with strategies to enhance NK cell function, including allogeneic NK cell therapy, may provide a synergistic approach to more effectively clear senescent cells, potentially leading to more profound rejuvenation and longevity effects (241).

By targeting metabolic pathways influenced by mTORC1, researchers have identified opportunities to augment NK cell-mediated tumor control, presenting new therapeutic avenues for treating age-related diseases and cancer. One study reveals that IL-10 significantly elevates IFN-γ production in expanded NK cells by approximately 18-fold, highlighting the critical role of mTORC1 in NK cell activation and suggesting that modulating metabolic pathways could improve NK cell responses in therapeutic settings (242). Wang et al., demonstrated that cytokine stimulation activates NK cells through metabolic reprogramming, increasing both glycolysis and mitochondrial oxidative phosphorylation (243). Poznanski et al., discussed the metabolic regulation of NK cell effector functions, identifying mTORC1 as a critical regulator of NK cell metabolic reprogramming. The study suggests that targeting mTORC1-dependent pathways could enhance NK cell effector function, offering potential therapeutic targets for immune interventions (244). The regulatory role of mTORC1 in NK cell metabolic reprogramming provides a valuable target for enhancing NK cell-mediated tumor control and immune responses. Research exploring the KLRG1-AMPK pathway provides insights into its role in NK cell function, particularly in the context of aging and cancer. The accumulation of KLRG1 bright NK cells, which are more prevalent in older individuals, is associated with the activation of AMPK. This association indicates a potential mechanism through which aging may impact NK cell activity and points to the KLRG1-AMPK pathway as a target for rejuvenating NK cell in aged populations.

Most cancers are known to impair the functionality of NK cells, which reduces the effectiveness of cell-based therapies for solid tumors. The mechanisms by which cancers diminish NK cell response are not fully understood, making it a key objective of immunotherapy to overcome this resistance. In a recent report, Portale et al. identified autophagy as a crucial regulator of NK cell anti-tumor activity. Using preclinical models of prostate cancer (PCa), they demonstrated that autophagy is essential for the tumor-killing capabilities of NK cells (245). Their findings suggest that cancer cells suppress the autophagic response by activating the CXCR4-C/EBPβ pathway in tumor-infiltrating NK cells, thereby undermining their ability to combat tumors. Furthermore, the study indicates that reactivating the autophagic pathway through pharmacological treatments or genetic modifications can restore the functional deficits of NK cells within tumors, enhancing their effectiveness in killing cancer cells in laboratory settings. This research highlights autophagy as an important intracellular checkpoint for NK cells and suggests that regulating autophagy could be a promising strategy to improve NK cell-based immunotherapies (245).

The enhancement of NK cell function through cytokine activation is a promising strategy for clearing pre-malignant senescent cells. Activation of NK cells with IL-2 has been shown to increase their ability to lyse tumor cells by enhancing perforin binding to the target cell membrane. IL-2 is pivotal for the proliferation, survival, and activation of NK cells, and it has been utilized in clinical settings to augment NK cell responses against tumor cells (246). IL-12, a pro-inflammatory cytokine, plays a critical role in the activation of NK cells, inducing the production of IFN-γ, which further stimulates the cytotoxic activity of NK cells against tumor cells (247). IL-15 has emerged as a potent stimulator, promoting their proliferation and sustained activation of NK cells without inducing the activation-induced cell death commonly seen with prolonged IL-2 exposure (248). IL-15’s role in supporting the development and function of memory-like NK cells, which exhibit enhanced responsiveness upon re-stimulation, represents a significant advancement in NK cell-based therapies (249). Moreover, the combination of IL-18 with IL-12 or IL-15 has been reported to synergistically enhance NK cell activation and improve their anti-tumor activity. IL-18 contributes to NK cell activation by upregulating the expression of perforin and granzymes, and when used in conjunction with IL-12 or IL-15, it can significantly boost NK cell-mediated cytotoxicity against various cancer cell lines (250). Among these cytokines, IL-15, an essential cytokine for NK cell proliferation and activation, is being tested in clinical trials to enhance NK cell responses in older adults and cancer patients (251).

Besides the enhancement of endogenous NK cells, allogeneic NK cell therapy for senescence has recently drawn more and more attention. Allogeneic NK cells from different sources: peripheral blood NK cells from young, healthy donors (252), CB NK cells (253), NK92 cell line (254), CB CD34+ derived NK cells (255), placenta-derived NK cells (256), and iPSC derived NK cells (257) could address the immune aging of endogenous NK cells. Recently, Celularity reported *in-vitro* data demonstrating placental hematopoietic stem cells (CD34+) derived unmodified NK cells (CYNK-001) can preferentially eliminate chemotherapy drugs induced senescent non-small cell lung carcinoma tumor cell line A549 cells compared to non-treated A549 cells (258). In the same study, the authors improved the persistence and efficacy of CYNK-001 cells by ectopically expressing soluble IL-15 and a high IgG binding affinity and protease cleavage-resistant CD16 variant (CYNK-201). The genetically modified CYNK-201 also showed higher killing of drugs-induced senescent A549 than non-treated A549, suggesting the potential of allogeneic NK cell therapy to remove senescent cells. These innovative approaches demonstrated the potential of allogeneic NK cell therapy, not only in removing senescent cell accumulation but also in rejuvenating the aging immune system.

**Figure 3:**
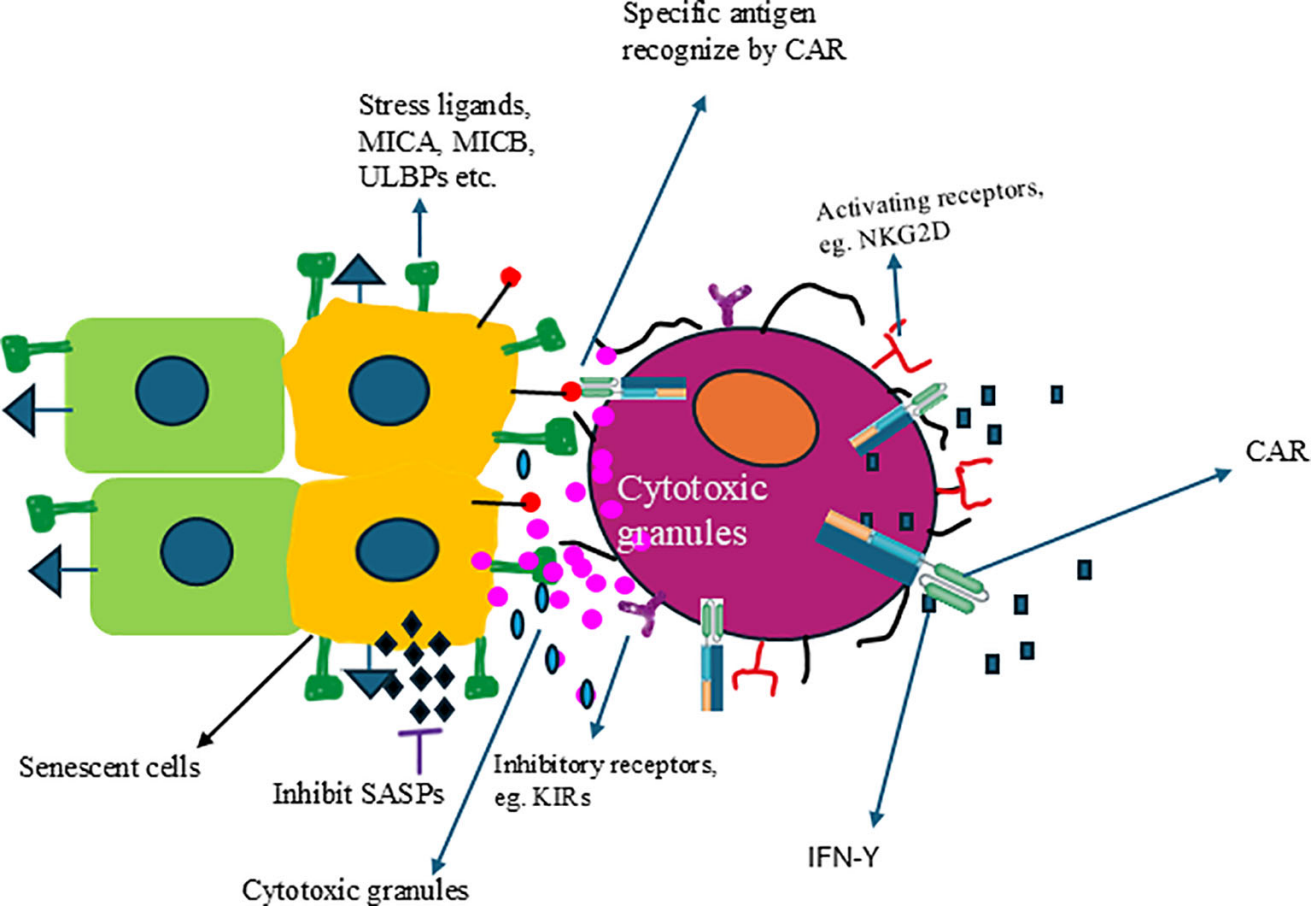
Engineered NK cells can specifically target and eliminate senescent cells. NK cells expressing CAR can recognize specific antigens expressed on the surface of senescent cells, resulting in the activation of NK cells and the release of cytolytic granules and kill the senescent cells.

Furthermore, methods already successfully utilized in cancer treatment, such as chimeric antigen receptor (CAR) and monoclonal antibodies, can also be applied to the NK cell platform. Compared to the autologous CAR-T cells approach, allogeneic CAR-NK cells might be a valuable tool for senescence immune surveillance with off-the-shelf, no GvHD, better safety profile clinically, and cost-effectiveness. The safety and efficacy of CAR-NK have been demonstrated using CB-derived NK cells expressing anti-CD19 chimeric antigen receptor and interleukin-15 (CAR19/IL-15) in 37 patients with CD19^+^ B cell malignancies (259). **Figure 3** illustrates how genetically engineered allogeneic CAR-NK cells can specifically eliminate senescent cells but not healthy cells. Multiple CAR targets have been evaluated under preclinical autologous CAR-T cell space, which could be applied in the allogenic CAR-NK cell field. Following the identification of NKG2D ligands upregulation in senescent cells, Yang et al., have shown that NKG2D-CAR T cells can eliminate senescent cells in aged mice and nonhuman primates (260). It was reported that high expression of fibroblast activation protein (FAP) in active cardiac fibroblasts drives abundant cardiac fibrosis and consequent myocardial disease. Adoptive transfer of FAP CAR-CD8^+^ T cells results in a significant reduction in cardiac fibrosis and restoration of function after injury in mice (261). The expression of Urokinase-type plasminogen activator receptor (uPAR), a cell-surface protein, is highly upregulated during senescence (262). uPAR-specific CAR T cells can safely eliminate senescent cells in young and aging mice. Treatment with anti-uPAR CAR-T cells improves exercise capacity in physiological aging. It ameliorates metabolic dysfunction (for example, improving glucose tolerance) in aged mice and mice on a high-fat diet (262, 263). While CAR-NK cells have not yet been tested for the removal of senescent cells, the CAR technology used to create CAR-T cells can also be utilized for CAR-NK cells. Furthermore, there should be a sustained effort to identify new antigens to develop more effective CAR-NK cells specifically designed for targeting and clearing senescent cells. One of the new targets could be DPP4-CAR NK cells. DPP4 (dipeptidyl peptidase 4) protein is selectively expressed on the surface of senescent, but not proliferating, human diploid fibroblasts (264). Higher DPP4 expression was found in peripheral blood samples from older subjects (78–88 years of age) compared to younger subjects (27–36 years of age), and this expression was more associated with monocytes and lymphocytes. Silencing the gene encoding DPP4 in these cells reduced the senescent phenotype, with decreased expressions of p21, p53, and p16 and increased expression of sirtuin1 (264). DPP4 is related to the cell surface expression of caveolin 1, which activates NF-κB and produces SASPs. Thus, DPP4-CAR NK cells could be a new therapeutic for senolysis.

Allogeneic NK cell therapy offers several advantages, including the potential for off-the-shelf safety, cost effectiveness, and the use of NK cells from young, healthy donors with optimal functional capacity. However, several challenges must be addressed to realize the full potential of NK cell therapy. First, the major barrier to NK cell therapy is the lack of persistence *in vivo* in the absence of cytokine support. While the short-term persistence in patients is desirable for safety, it is a limiting factor for efficacy. NK cell engineering strategies incorporate genes to ectopically express cytokines such as IL-15 to improve persistence (265). Another challenge is graft rejection by the host’s immune system. To protect allogeneic NK cells from being rejected, scientists are using stealth techniques, including knocking down MHC Class I and Class II molecules and overexpressing HLA-E/CD47 (266). Additionally, NK cells are vulnerable to damage during the freeze-thaw process. NK cells are susceptible to the freeze-thaw process. Thawing procedures significantly reduce the survival rate and cytotoxic capabilities of NK cells, but functional activity may be restored by incubation with interleukin-2 (IL-2) (267). Therefore, developing an optimal cryopreservation and recovery protocol is essential for NK cell therapy to become a viable “off-the-shelf” treatment. Senolytic drugs represent a crucial component of the therapeutic arsenal against cellular senescence, offering a direct means to reduce the senescent cell burden. The combination of senolytic drugs and adoptive NK cell therapy could provide a comprehensive approach to combat the deleterious effects of aging and improve overall healthspan.

## 6 Conclusions and outlook

Aging is a universal process associated with an increased risk of various diseases, including Alzheimer’s, cardiovascular diseases, and cancers. Preventing aging at its source offers the prospect of extending healthy, productive years, significantly enhancing individuals’ quality of life. By reducing the prevalence of age-related diseases, individuals can maintain independence and physical and cognitive function well into later life, benefiting society by preserving knowledge and experience. The intricate relationship between NK cell senescence and cancer highlights the dual roles of cellular senescence in tumor suppression and promotion, particularly within the aging immune system. Immunosenescence leads to a decline in NK cell function, contributing to increased vulnerability to infections and malignancies in older adults. Understanding the mechanisms that drive NK cell senescence and their impact on cancer progression is crucial for developing effective therapeutic strategies. Adoptive NK cell therapy emerges as a promising approach, potentially rejuvenating the immune surveillance system by targeting both malignant and senescent cells. Recent advances in biotechnology and medicine have offered the possibility of preventing or significantly delaying the aging process rather than merely treating its associated diseases. Further research into senolytic drugs and the enhancement of NK cell function could provide synergistic effects, enhancing the clearance of senescent cells and improving outcomes for age-related diseases. Addressing these challenges through innovative therapeutic strategies holds the promise of extending healthspan and improving the quality of life for the aging population.
